# Comparison of 3 assessment modes of acupuncture effect on patients with chronic prostatitis/chronic pelvic pain syndrome: A study protocol for a randomized controlled trial

**DOI:** 10.1097/MD.0000000000012887

**Published:** 2018-10-19

**Authors:** Jing Zhou, Yan Liu, Chunbin Li, Zhishun Liu

**Affiliations:** a Department of Acupuncture, Guang’anmen Hospital, China Academy of Chinese Medical Sciences; b China Academy of Chinese Medical Sciences; c Institute of Basic Research in Clinical Medicine, China Academy of Chinese Medical Sciences, Beijing, China.

**Keywords:** acupuncture, chronic prostatitis/chronic pelvic pain syndrome, modes of assessment, protocol, sham-acupuncture

## Abstract

**Background:**

Chronic prostatitis/chronic pelvic pain syndrome (CP/CPPS) is a common disorder. Some studies have indicated that acupuncture may ameliorate the symptoms of CP/CPPS. However, results are varied and range widely, perhaps due to different modes of assessment including timepoints, places of assessment, and especially subjective scales. We propose to determine the efficacy of acupuncture relative to sham acupuncture for patients with CP/CPPS, and compare different modes of assessment regarding the therapeutic effects of acupuncture.

**Methods:**

Sixty patients with CP/CPPS will be randomly assigned to receive either acupuncture or sham-acupuncture (30 patients, each). Treatment will be conducted 3 times/week, for 4 weeks. The coprimary outcomes will each be the change from baseline of the total NIH (National Institutes of Health) CPSI (Chronic Prostatitis Symptom Index) score associated with 3 modes of assessment: Mode 1, the scale recorded at the hospital within 10 minutes after the last session of 4 weeks of acupuncture treatment, in the company of the outcome assessors; Mode 2, the scale recorded the same day, but not at the hospital; and Mode 3, the scale recorded at the hospital 1 to 3 days after the last acupuncture session. The 3 key secondary outcomes include will be the 3 modes assessment of the changes from baseline of the NIH-CPSI total scores in the acupuncture group at week 4 after treatment. Analysis was by intention-to-treat, and multiplicity was controlled for with a step-down closed-testing procedure.

**Discussion:**

To our knowledge, previous studies did not include the details of assessment scales when determining the efficacy of acupuncture in CP/CPPS. Furthermore, it is not clear which mode was used to assess the effect. The answers provided by the proposed study may improve the reproducibility of acupuncture research and the reliability of results.

## Abstract 2

Supplemental Digital Content is available in the text

## 1 Introduction

Chronic prostatitis/chronic pelvic pain syndrome (CP/CPPS) is a common disorder with 4 domain manifestations: urogenital pain, lower urinary tract symptoms, psychological issues, and sexual dysfunction.^[1]^ Men of all ages and races may experience prostatitis, with a worldwide prevalence of 2% to 10%,^[2]^ while the prevalence is 15% to 16% in Asia, Europe, and North America.^[3]^ More than 90% of patients with symptomatic prostatitis have CP/CPPS.^[4]^ The first-line treatments for CP/CPPS include antibiotics, α-adrenergic antagonists, and simple analgesics.^[5]^ However, due to the multifactorial nature of this syndrome, no single therapeutic option is adequate and multimodal therapies are needed.^[6,7]^

Currently, there are many management approaches for CP/CPPS.^[8]^ According to previous studies, acupuncture has shown promise for the amelioration of CP/CPPS symptoms, particularly for relieving pain.^[9–12]^ The National Institutes of Health (NIH) Chronic Prostatitis Symptom Index (CPSI)^[13]^ is a valid and easily self-administered outcome measure for men with chronic prostatitis. Our previous pilot study^[14]^ showed that after 8 weeks of treatment, acupuncture was associated with a clinically significant decrease in the NIH-CPSI total score. However, in recent studies the change from baseline of the NIH-CPSI after acupuncture treatment has ranged widely,^[15–21]^ and higher than the minimal clinically important difference of the NIH-CPSI is 4 points.^[22,23]^ The results of these studies have been highly variable.

The primary presentation of CP/CPPS is urological pain,^[4]^ and pain is a predominant topic of research in acupuncture.^[24]^ Studies have shown that acupuncture has an immediate pain-relieving effect.^[25]^ The range of results of previous studies for the efficacy of acupuncture treatment in CP/CPPS may be due to differences in timepoints, places of assessment, and especially the subjective scales used for assessment.

### 1.1 Objective of this study

This study will determine the efficacy of acupuncture treatment for patients with CP/CPPS after 4 weeks of treatment, and evaluate 3 modes of assessment of efficacy.

## 2 Methods

### 2.1 Study design

This trial is designed as a randomized sham-controlled multicenter trial with 2 parallel groups. It will be conducted at the following 5 hospitals: Guang’anmen Hospital, West China Hospital of Sichuan University, Yantai Hospital of Traditional Chinese Medicine, Hengyang Hospital Affiliated to Hunan University of Chinese Medicine, and The First Hospital of Hunan University of Chinese Medicine. The development of the study protocol conformed to the guidelines of the Standard Protocol Items: Recommendations for Interventional Trials (SPIRIT)^[26]^ and the Standards for Reporting Interventions in Clinical Trials of Acupuncture (STRICTA).^[27]^ This study will be conducted in accordance with the principles of the Declaration of Helsinki^[28]^ and has been approved by the Ethics Committee of Guang’anmen Hospital (Ethical number 2018-098-KY, see Table 1, Supplemental Content, which illustrates the ethical documentation) and the Research Ethics Committee of the aforementioned hospitals.

This trial has been registered at www.clinicaltrials.gov (NCT 03641807).

### 2.2 Randomization and blinding

**Figure 1:**
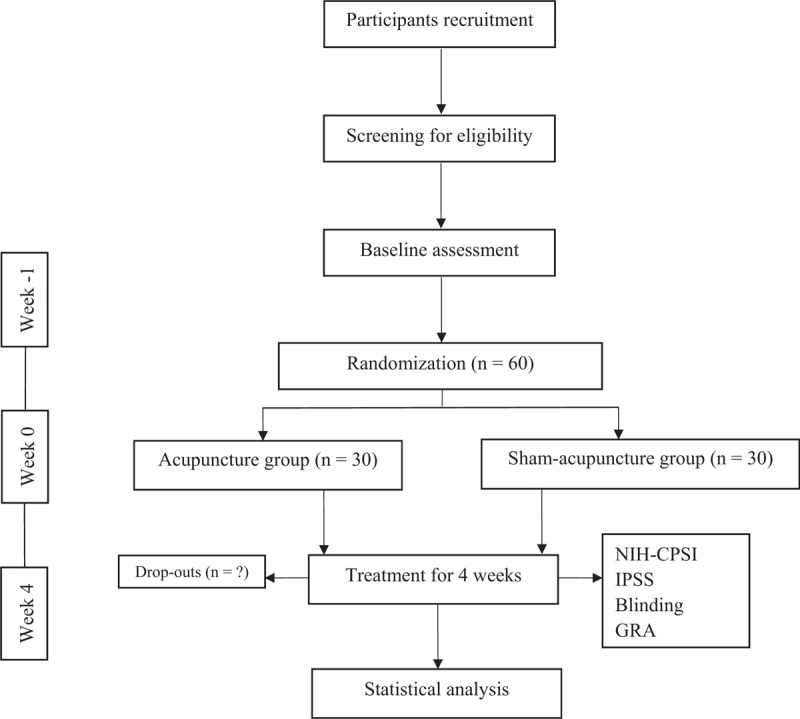
Flowchart of the trial.

**Figure 2:**
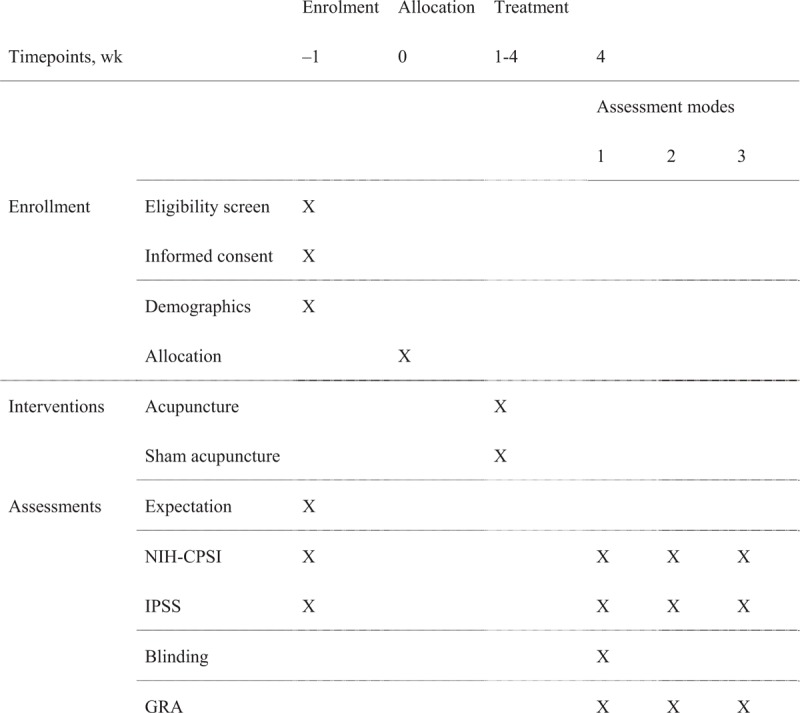
Standard Protocol Items: Recommendations for Interventional Trials (SPIRIT).

All the subjects of this study will fulfill the inclusion criteria and provide written informed consent before enrollment. The enrollees will be randomly assigned, by computerized simple random sampling, to receive either acupuncture or sham acupuncture at a population ratio of 1:1. The allocation sequence will be generated by the Institute of Basic Research in Clinical Medicine affiliated to China Academy of Chinese Medical Sciences. The participants, outcome assessors, and statisticians will be blinded to the allocation. The flowchart is shown in Fig. 1 and the timepoints of enrolment, interventions, and assessments are shown in Fig. 2.

### 2.3 Study population and recruitment

Sixty participants will be recruited from September 2018 to September 2019 through posters, hospital websites, and networks. Recruitment will be conducted by research assistants.

Each participant will have a medical history, physical examination, prostatic fluid cultures, prostate-specific antigen evaluation, urine flow rate, and residual urine examination. Microbiology of the prostatic fluid/urine specimen will be collected by the 2-glass test.^[29]^ A urologist will be responsible for diagnosis.

The criteria for inclusion in this study are the following: aged 18 to 50 years; with CP/CPPS, diagnosed according to the NIH CP/CPPS consensus (discomfort or pain in the pelvic region ≥3 months during the previous 6 months^[4]^); and NIH-CPSI total score ≥15. Potential participants will be excluded for any of the following: urologic disease; residual urine volume ≥100 mL, Qmax ≤15 mL/s; use of 5-alpha reductase inhibitor, alpha-blockers, antibiotics or any other prostatitis-specific medication during the previous 1 month; diseases that influence urologic symptoms; or any acute disease or severe disease requiring treatment. Urologic diseases that will prevent inclusion are bladder outlet obstruction, overactive bladder, neuropathic bladder, interstitial cystitis, cystitis glandularis, bladder cancer, acute prostatitis or bacterial prostatitis, benign prostatic hyperplasia, prostate cancer, symptomatic urinary tract infection, and organic diseases of urinary system. Diseases that influence urologic symptoms are multiple sclerosis, multiple system atrophy, stroke, Alzheimer disease, Parkinson's disease, spinal cord injury, cauda equina injury, and sexually transmitted disease.

### 2.4 Interventions

Participants in both the acupuncture and sham groups will be treated for 4 weeks, with 3 sessions per week, making 12 sessions. Treatment will be performed by acupuncturists who have an official license, with strict training related to the standardization and details of performing treatment. Two acupuncturists each will be responsible for the 2 groups. The use of drugs or other therapies for CP/CPPS will be avoided during the study period, unless the symptoms of participants are intolerable. The medications used should be recorded in detail.

#### 2.4.1 Acupuncture group

Development of the acupuncture protocol was based on the consensus of acupuncturists from Guang’anmen Hospital. The locations of the acupoints are in accordance with the World Health Organization standard acupuncture point locations.^[30]^ Bilateral Shenshu (BL23), Zhongliao (BL33), Huiyang (BL35), and Sanyinjiao (SP6) will be inserted using Hwato-brand (Suzhou Medical Appliance Factory, China) disposable acupuncture needles (sizes 0.30 × 75 and 0.30 × 40 mm). With patients prone, after routine sterilization, bilateral Zhongliao (BL33) will be inserted to a depth of 50 to 60 mm with a 30° to 45° angle in an inferomedial direction using needles (0.30 mm in diameter, 75 mm in length). Bilateral Huiyang (BL35) will be inserted to a depth of 50 to 60 mm with a slightly superolateral direction using needles (0.30 mm in diameter, 75 mm in length). Bilateral Shenshu (BL23) and Sanyinjiao (SP6) will be inserted vertically to a depth of 25 to 30 mm using needles (0.30 mm in diameter, 40 mm in length). Manipulation of the needles by lifting and thrusting combined with twirling and rotating evenly will be performed until deqi occurs, defined as a sensation of soreness, numbness, heaviness, and ache.^[31]^ Manipulations will be applied every 10 minutes and each session will last for 30 minutes.

#### 2.4.2 Sham acupuncture group

Bilateral sham BL 23, BL 33, BL 35 (15 mm to BL23, BL33, and BL35) and SP6 (10 mm to SP6) will be inserted by needles (0.20 mm in diameter, 25 mm in length) to a depth of 2 to 3 mm without manipulation.

### 2.5 Outcome measurement

The 3 coprimary outcomes are the change from baseline in total NIH-CPSI score measured respectively by the 3 assessment modes after the 4-week treatment.^[13]^ The NIH-CPSI has a total possible score of 43; higher scores indicate more severe symptoms. The 9 items of this scale are stratified into 3 domains as follows: pain/discomfort (location/type, frequency and severity, 0–21 points); urinary symptoms (10 points); and quality of life (0–12 points). The minimal clinically important difference of the NIH-CPSI is 4 points.^[4]^ The 3 modes of assessment are as follows: Mode 1, the scale is recorded at the hospital within 10 minutes after the twelfth (last) treatment of the 4-week treatment period, in the company of the outcome assessors; Mode 2, the scale is recorded the same day, but not at the hospital; and Mode 3, the scale is recorded at the hospital 1 to 3 days after the last acupuncture session. The outcome assessors will be responsible for calling the participants by telephone to remind them to fill the scale after leaving the hospital, and recording the accurate time for filling the scale.

The 3 key secondary outcomes are the 3 modes of assessment of the changes from baseline of the NIH-CPSI total score in the acupuncture group.

The secondary outcomes of this study are the changes from baseline of subscale scores of the NIH-CPSI, and the International Prostate Symptom Score (IPSS). The IPSS (Hong Kong Chinese version 2) is a valid, reliable, and sensitive measure to assess Chinese males, with 7 questions concerning urinary symptoms and 1 question concerning quality of life.^[32]^ The total possible IPSS score is 35 (asymptomatic to very symptomatic). Symptoms evaluated by the IPSS are categorized as mild (0–7), moderate (8–19), or severe (20–35).

The proportions of participants in each response category of the Global Response Assessment (GRA) will be assessed. The GRA comprises 7 response categories: markedly worsened, moderately worsened, slightly worsened, no change, slightly improved, moderately improved, and markedly improved.

The participants’ expectations regarding acupuncture will be measured at baseline. Participants will answer 2 questions: “In general, do you believe acupuncture is effective for treating the illness?”; and “Do you think acupuncture will be helpful to improve your CP/CPPS symptoms?” Participants will choose “Unclear,” “Yes,” or “No” as the answer.

In addition, during the fourth week of the study period (sessions 11 or 12) participants will be asked to answer the following question within 5 minutes after treatment: “Do you think you have received traditional acupuncture in the past weeks?” The participants will be able to choose one of the following options as the answer: “Unclear,” “Yes,” or “No.”

### 2.6 Safety assessment

Adverse events related to acupuncture treatment and the rate of incidence will be carefully recorded in the case report forms. Adverse events include severe pain, broken needle, fainting, local hematoma, localized infection, and postacupuncture discomfort. Severe pain will be reported by Visual Analogue Scale (≥7 points). Postacupuncture discomfort may be nausea, vomiting, palpitation, dizziness, headache, anorexia, and insomnia during treatment period. The severity and duration of postacupuncture discomfort will also be recorded.

In addition, adverse events that are irrelevant to the treatment will be recorded during the study period. In the event of any serious adverse event, researchers will report the details to the principal investigator (ZL) and the Medical Ethics Committee within 24 hours.

### 2.7 Calculation of sample size and statistical analyses

We hypothesize that acupuncture will be more effective than sham acupuncture, according to at least one assessment mode, at week 4 after treatment. Based on the results of a previous electroacupuncture study,^[14]^ we estimated that 28 patients were required in each group to provide 90% power to detect a difference of 4 in the NIH-CPSI total score, with a standard deviation of 4.5 at a 2-sided alpha level of 0.05. Accounting for a 6% loss during follow-up, we calculated that we would need to enroll 60 patients.

All efficacy analyses were performed on an intention-to-treat population, and included all patients who were randomly assigned, regardless of whether they received treatment. Safety analyses were defined as all patients who received at least one treatment session. We analyzed the change from baseline in the NIH-CPSI total score by fitting a general linear model, with the baseline value as a covariate, and treatment as a fixed effect. The same approach was used for the 3 key secondary outcomes and the change from baseline in the IPSS score. Categorical variables were compared using Fisher exact test or the Wilcoxon rank-sum test, as appropriate.

To account for multiplicity to test the 3 primary hypotheses and the 3 secondary hypotheses, a hierarchical procedure for multiple testing was used to control the overall type I error rate. For further details, see Table 2, Supplemental Content, which illustrates the Hierarchical procedure for multiple testing, in 6 steps.

Missing data on the primary outcome were assumed due to random occurrence and were imputed using the multiple imputation method. All statistical analyses were performed using Statistics Analysis System version 9.4 software (SAS Institute) with 2-sided tests at a significance level of 0.05.

### 2.8 Quality control

To ensure the quality of this study, all researchers will receive training related to the entire process of this trial. The procedure of acupuncture will be based on strict standards and performed by acupuncturists with at least 2 years clinical experience. An independent third party will supervise the entire study.

## 3 Discussion

This proposed study will assess the efficacy of acupuncture after 4 weeks of treatment for patients with CP/CPPS, and 3 modes of evaluation of acupuncture efficacy. To our knowledge, almost none of the other recent studies have reported the details of assessment when using scales as the primary outcomes, and it is not clear in which mode the effect of acupuncture was assessed. The objective of the present study proposed here is to evaluate modes of assessment, to improve the repeatability of acupuncture research, and improve the reliability of results.

The results of our previous pilot study^[14]^ showed that, after 8 weeks of treatment, the symptoms of patients with CP/CPPS were clinically improved. However, there is no exact and definitive means of assessment in practical clinical acupuncture research. For example, among studies the timepoints of assessment have varied between immediately or a few days after acupuncture, and the assessment scales may be recorded within or outside the hospital.

In general, acupuncture ameliorates pain immediately and the effect lasts for a few hours.^[33,34]^ However, assessments conducted immediately or soon after treatment may exaggerate the results. In addition, the influence which patients are accompanied with outcome assessors on objective effect assessment cannot be ignored. Meanwhile, personal and situational factors which may affect treatment response^[34]^ should be considered.

In this study, the efficacy of acupuncture will be evaluated at week 4 using 3 assessments modes. Practically however, it is difficult to restrict the time interval for recording any of these too strictly. Hence, the exact time of assessment will be recorded. The timepoint of mode 3 will be regarded as the longest time interval to assess the effect of week 4.

This study has 3 main limitations. Firstly, 4 weeks of treatment with 12 sessions may not be enough for patients, and assessing the efficacy at week 4 will result in some bias. However, this is a preliminary study meant to provide some indications for future acupuncture research when using scales as primary assessment tools. Secondly, it is difficult to blind the acupuncturists to the treatment group, which may bring some bias. Finally, the sample size is small. In future studies, larger sample sizes will be needed and more timepoints will be included within the assessing modes to monitor dynamic changes associated with acupuncture in CP/CPPS.

## Acknowledgment

The authors sincerely thank the editors of Medjaden Bioscience Limited for their help proofreading the manuscript.

## Author contributions

JZ, YL, and ZL conceived the concept and design of the study. JZ and YL drafted and edited the final paper for submission. ZL reviewed and amended the final paper. CL prepared the related information sheets, consent forms, and case report forms. All authors approved the final manuscript.

**Conceptualization:** Jing Zhou, Yan Liu, Zhishun Liu.

**Funding acquisition:** Zhishun Liu.

**Investigation:** Chunbin Li.

**Methodology:** Jing Zhou, Yan Liu, Zhishun Liu.

**Project administration:** Zhishun Liu.

**Resources:** Chunbin Li.

**Supervision:** Zhishun Liu.

**Validation:** Jing Zhou, Yan Liu, Zhishun Liu.

**Writing – original draft:** Jing Zhou, Yan Liu.

**Writing – review & editing:** Jing Zhou, Yan Liu.

Zhishun Liu orcid: 0000-0001-7570-8917.

## Supplementary Material

Supplemental Digital Content
