# Supplementary material for: Comparison of 3 assessment modes of acupuncture effect on patients with chronic prostatitis/chronic pelvic pain syndrome: A study protocol for a randomized controlled trial
Source: Medicine (Baltimore). 2018 Oct 19;97(42):e12887. doi: 10.1097/MD.0000000000012887 (PMC6211918; doi:10.1097/MD.0000000000012887)
Supplement: Supplemental Digital Content [file medi-97-e12887-s001.doc]

**Supplement Table 1. Ethical Approval Documentation**

| **Title of the study** | Comparison of three assessment modes of acupuncture effect on patients with chronic prostatitis/chronic pelvic pain syndrome: a randomized controlled trial | | | |
| --- | --- | --- | --- | --- |
| **Ethics approval number** | 2018-098-KY-01 | | | |
| **Research Unit** | Guang’anmen Hospital, China Academy of Chinese Medical Sciences | | | |
| **Major researcher** | Zhishun Liu | **Department of the researchers** | Department of Acupuncture | |
| **Category of the ethical review of this study** | Review | **Method of the ethical review of this study** | Meeting | |
| **Date of the ethical review of this study** | July 20th, 2018 | **Place of the ethical review of this study** | The meeting room of Guang’anmen Hospital | |
| **Name of Review members** | Xinghua Feng and Lizhen Gu | | | |
| **Approval Documents** | 1. Protocol of this study  2. Informed consent  3. Case Report Form  4. Recruitment advertisement  5. Researcher Handbook | | | |
| **Reviewers’ Comments:** | According to the ‘Ethical Review of Biomedical Research Related to Human’ formulated by Ministry of Public Health, ‘Specifications for the quality control of drugs used in clinical trials in China Academy of Chinese Medical Sciences’ formulated by State food and drug administration, ‘The Provisions for Medical Device Classification’, ‘Guidelines for ethical review of clinical trials related to drugs’, ‘Standard for clinical research on ethical review and management of TCM clinical research’ formulated by State Administration of Traditional Chinese Medicine(under the Ministry of Public Health), ‘Declaration of Helsinki’, ‘International Moral Guide for biomedical research’ formulated by Council for International Organizations of Medical Sciences, all the reviewers approved the conduction of this study.  Please follow the principle of GCP and protocol of this study approved by the review boards and ethics committees and protect the rights of participants during the process of this study.  If the starting time of the study is later than validity date of the ethical document, the ethical approval will be invalid, and researchers should resubmit an application for ethical approval.  If the major researcher has been altered or any amendments in the protocol, informed consent and recruitment advertisement have been made, the researchers should resubmit an application for ethical approval.  If there are any severe adverse events or adverse events affecting the benefit of the research, the researchers should submit the report of adverse events in 15 days and submit the report of adverse events which cause death in 7 days.  Researchers should submit report of progress of the study before one month of the deadline according to frequency of ethical review. Researchers should submit summary reports about circumstances severely affecting the ongoing of the study and increasing the risk of participants in time to review boards and ethics committees.  If the following circumstances happen in the whole process of this study, applicants/researchers/ inspectors should submit a report about reasons causing the violation of study design: 1. Participants who met the inclusion criteria. 2. Participants who met the provisions of termination but weren’t terminated. 3. Participants who were given incorrect therapy or wrong doses of medication. 4. Participants who had taken forbidden combined medication according to the design of the study. 5. Any conditions which violating the principle of GCP and affecting the participants’ rights/health or the scientific nature of the study.  Please submit a report in time when the study is finished or terminated in advance. | | | |
| **Period of validity of this ethical approval document** | From July 24th, 2018 to July 23th, 2019 | | | |
| **Frequency of ethical review** | Every 12 months | | | |
| **Contact information** | **Name:** Jie Qiao | | | |
|  | **Phone:** 86-010-88001552 | | | |
|  | **Email:** gamhec@126.com | | | |
| **Signature of Chairman of the review boards and ethics committees** | | | | Haibo Yin |

**Article title:** Comparison of three assessment modes of acupuncture effect on patients with chronic prostatitis/chronic pelvic pain syndrome: a study protocol for a randomized controlled trial.

**First author:** Jing Zhou and Yan Liu.

**Supplement Table 2.** Hierarchical procedure for multiple testing, in 6 steps *.

| 1 | Acupuncture would be more effective than sham acupuncture on Mode 1 |
| --- | --- |
| 2 | Acupuncture would be more effective than sham acupuncture on Mode 2 |
| 3 | Acupuncture would be more effective than sham acupuncture on Mode 3 |
| 4 | Mode 1 would be more effective than Mode 3 in the acupuncture group |
| 5 | Mode 2 would be more effective than Mode 3 in the acupuncture group |
| 6 | Mode 1 would be more effective than Mode 2 in the acupuncture group |

* If any of the steps failed to meet statistical significance, the testing procedure would be stop and subsequent tests would not be performed.

**Article title:** Comparison of three assessment modes of acupuncture effect on patients with chronic prostatitis/chronic pelvic pain syndrome: a study protocol for a randomized controlled trial.

**First author:** Jing Zhou and Yan Liu.
